# Elucidating Development Trajectories of Brain Functional Abnormalities in Major Depressive Disorder Utilizing a Data‐Driven Disease Progression Model

**DOI:** 10.1002/hbm.70249

**Published:** 2025-06-04

**Authors:** Yuhong Zheng, Peng Wang, Chi Yao, Jinghua Wang, Jinhui Wang, Shao‐Wei Xue

**Affiliations:** ^1^ Center for Cognition and Brain Disorders/Department of Neurology The Affiliated Hospital, Hangzhou Normal University Hangzhou Zhejiang China; ^2^ Institute for Brain Research and Rehabilitation, South China Normal University Guangzhou China

**Keywords:** amplitude of low‐frequency fluctuations, cognitive terms, disease progression trajectories, functional magnetic resonance imaging, gray matter volume, major depressive disorder

## Abstract

Concerns have arisen regarding the heterogeneity of patients with major depressive disorder (MDD), particularly when the varying disease progression trajectories among individuals are overlooked. Recognizing these distinct trajectories is crucial for personalized assessments and accurate disease progression predictions in MDD, posing a significant challenge in clinical practice. We utilized a data‐driven subtype and stage inference (SuStaIn) model to infer trajectories based on cross‐sectional amplitude of low‐frequency fluctuations (ALFF) derived from resting‐state functional magnetic resonance imaging data of 833 patients with MDD and 834 healthy controls. Based on distinct trajectories, two subtypes of MDD were identified: Subtype 1 showed declining ALFF from paracentral lobule (PCL) to thalamus to medial orbitofrontal cortex (OFCmed), with higher core depression scores and gray matter atrophy, whereas Subtype 2 had an opposing trajectory, with initial OFCmed ALFF decrease gradually extending to PCL. Our findings contribute to a better understanding of MDD heterogeneity and facilitate precise disease progression predictions.

## Graphical abstract

Major depressive disorder exhibits profound phenotypic and temporal heterogeneity. This study aims to identify distinct brain change trajectories in MDD using the subtype and stage inference model to deepen our understanding of heterogeneity, as well as to compare the cognitive function change patterns among subtypes with different trajectories.

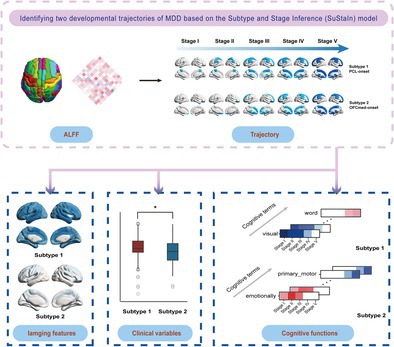

## 1 Introduction

Major depressive disorder (MDD) is a prevalent, chronic, and debilitating mental health condition affecting approximately 350 million people worldwide (James et al. 2018), posing a significant challenge to both public health and societal well‐being (Marx et al. 2023). The complexity of MDD is further compounded by the extensive phenotypic heterogeneity observed across its clinical manifestations. This heterogeneity manifests in a diverse array of symptoms, leading to a spectrum of presentations among patients with MDD ranging from mild to severe (Lin et al. 2023). Numerous studies have extensively documented these subtype differences, underscoring the urgent need for a deeper comprehension of MDD (Chen, Wang, et al. 2023; Sun, Sun, et al. 2023; Wang et al. 2021). For instance, some patients primarily exhibit psychological symptoms, including persistent sadness, loss of interest, and feelings of guilt or worthlessness (Marx et al. 2023). Others may display predominantly physical symptoms, such as chronic fatigue, insomnia, and alterations in appetite and weight (Liu, Tu, et al. 2021). Additionally, cognitive symptoms, such as difficulties with concentration, decision‐making, or memory, can vary considerably among individuals (Liu, Chen, et al. 2023). The recognition of phenotypic heterogeneity in MDD has significant implications for both research and clinical practice.

Moreover, an increasing body of research has shown that MDD progresses through distinct and identifiable stages (Maj et al. 2020). Even within the same individual, the clinical symptoms and severity of MDD can fluctuate significantly as the stages advance, further exacerbating phenotypic heterogeneity (Sun, Sun, et al. 2023; Wang et al. 2021; Liu, Tu, et al. 2021; Liu, Chen, et al. 2023; Maj et al. 2020; Verduijn et al. 2017; Zhang et al. 2023). This temporal heterogeneity underscores the need to consider both subtype classification and the temporal progression of the disorder when understanding the heterogeneity of MDD. This heterogeneity is not only reflected in symptoms but also in brain functional abnormalities. Multiple resting‐state functional magnetic resonance imaging (fMRI) studies have shown that changes in brain functional abnormalities in patients with MDD are closely related to the progression of clinical symptoms (Chen et al. 2021; Qiu et al. 2024; Zhu et al. 2023). For example, studies based on the amplitude of low‐frequency fluctuations (ALFF) have demonstrated that MDD subgroups primarily characterized by somatic symptoms exhibit reduced ALFF in the sensorimotor network (SMN) (Liu, Tu, et al. 2021), whereas subgroups with predominantly gastrointestinal symptoms show more significant abnormalities in regions such as the prefrontal cortex (Fu et al. 2021). Additionally, a longitudinal study revealed a persistent reduction in functional activity in the precuneus and posterior cingulate cortex in patients with MDD (Wang et al. 2020), suggesting significant fluctuations in functional abnormalities over time. These findings indicate that the complex functional heterogeneity of MDD is linked to its clinical heterogeneity (phenotype and temporal). It also highlights the importance of adopting a comprehensive and individualized approach to accurately diagnose and effectively treat MDD, taking into account each patient's unique symptom profile and clinical history. However, most studies have analyzed MDD heterogeneity solely based on differences between subtypes, with limited examination of heterogeneity in conjunction with disease progression (Chen, Dai, et al. 2023; Estarellas et al. 2024). One plausible reason for this is that longitudinal studies, which are essential for capturing this temporal dimension, face challenges such as high costs, extended durations, and difficulties with participant retention (Gustavson et al. 2012; Teague et al. 2018). Consequently, researchers have turned to cross‐sectional data and unsupervised machine‐learning techniques to infer disease heterogeneity.

One such approach is the subtype and stage inference (SuStaIn) model, a data‐driven framework that utilizes cross‐sectional data to infer distinct trajectories of biomarker progression (Young et al. 2018). These trajectories elucidate the varying patterns of biomarker changes over time while simultaneously stratifying patients into distinct subgroups (subtypes), thereby addressing both dimensions of disease heterogeneity (Collij et al. 2022; Hong et al. 2023). SuStaIn has demonstrated its robustness and adaptability in conditions such as Alzheimer's disease (Vogel et al. 2021), schizophrenia (Jiang et al. 2023), and epilepsy (Jiang et al. 2024). For example, in Alzheimer's disease, SuStaIn has been employed to analyze multimodal imaging data, revealing subtypes characterized by distinct patterns of tau protein spread in the brain (Vogel et al. 2021). These subtypes have critical implications for disease staging, prognosis, and targeted interventions. The success of these studies underscores SuStaIn's capacity to capture subtle yet meaningful variations in disease trajectories, enhancing our understanding of diseases and fostering the development of precision medicine. Existing research has shown that patients with depression often exhibit widespread alterations in whole‐brain connectivity and excessive network integration (Kaiser et al. 2015; Yan et al. 2019). Functional abnormalities in localized brain regions may gradually spread to other areas through abnormal functional connections and network dysregulation, forming an aberrant dynamic progression. For example, a large‐scale resting‐state dynamic causal modeling (DCM) study found that patients with MDD exhibit a directional imbalance characterized by enhanced excitatory connections and weakened inhibitory connections within the default mode network (DMN) and between the DMN and the salience network (SN) (Li et al. 2020). This suggests that localized functional abnormalities may disrupt normal network communication and drive the spread of dysfunction along network pathways. Additionally, connectome gradient analysis has revealed a compression of the primary‐to‐high‐order network gradient in patients with MDD (Xia et al. 2022). This macro‐level integration abnormality may reflect patterns of functional dysfunction along the principal gradient axis (e.g., SMN to DMN) and, together with coordinated changes in subcortical nodes such as the thalamus (THA) and hippocampus (Xiao et al. 2023), constitutes a cortico‐subcortical spreading mechanism. The SuStaIn model, by capturing the spatiotemporal sequence of ALFF changes, provides a potential means to uncover possible abnormal dynamic progression patterns in MDD.

The primary objective of this study was to identify distinct brain change trajectories in MDD. We input cross‐sectional brain functional data from patients with MDD and healthy controls into the SuStaIn model to infer different disease progression trajectories. Based on these trajectories, we categorized patients into various subtypes and compared the clinical differences among them. Subsequently, we examined the relationship between different progression trajectories and patterns of cognitive function changes. We hypothesized that MDD encompasses distinct and unique brain functional change trajectories, which profoundly reflect the phenotypic and temporal heterogeneity of MDD. By elucidating the trajectories of functional alterations in MDD, this study aims to enhance our understanding of the heterogeneity of this disorder and provide a reference for assessing patients' current individualized states and predicting future developmental trends.

## 2 Methods

### 2.1 Participants

A total of 833 patients diagnosed with MDD (mean age: 35.7 ± 12.3 years, 64.3% female) and 834 healthy controls (HC, mean age: 35.6 ± 13.8 years, 59.7% female) were obtained from a publicly accessible dataset provided by the REST‐meta‐MDD project within the DIRECT consortium (Yan et al. 2019; Chen et al. 2022). The data for this project comes from 25 research sites in 17 hospitals and universities, including 1300 MDD and 1128 HC, and details of the project are provided in the Method S1. We excluded certain sample data based on the following criteria: (1) participants lacking demographic information were excluded; (2) participants who were either younger than 18 years or older than 65 years of age were excluded; (3) participants exhibiting excessive head motion, specifically defined as having a mean framewise displacement (FD) exceeding 0.2 mm and a maximum FD exceeding 2 mm, were disqualified; (4) patients with MDD whose scores on the 17‐item Hamilton Rating Scale for Depression (HAMD) fell below 8 points were also excluded; (5) participants with imaging distortions detected through rigorous visual inspection or with a loss of imaging signal within any brain regions used in this study, as defined by the automated anatomical labeling (AAL‐2) template, were excluded; (6) to balance maximizing the overall sample size while minimizing potential biases, we excluded data from sites where the number of patients with MDD or healthy control subjects fell below 10. Finally, this study screened 833 patients with MDD and 834 HC (from 18 sites) from the REST‐meta‐MDD project. The research procedures strictly adhered to the principles outlined in the Helsinki Declaration and were granted approval by the Institutional Review Boards of all participating research sites. Prior to their participation in the formal testing, all subjects provided written informed consent.

### 2.2 Data Preprocessing

The preprocessing of resting‐state functional magnetic resonance imaging (rs‐fMRI) and T1‐weighted MRI data adhered to the standardized protocols incorporated within the DPARSF software package. Specifically, the initial 10 functional volumes of the rs‐fMRI data were discarded to ensure the attainment of signal stability and adequate adaptation to the scanning environment. Subsequently, slice timing correction, realignment of head motion, spatial normalization, and temporal band‐pass filtering (0.01–0.1 Hz) were conducted. Friston‐24 motion parameters, white matter signal, and cerebrospinal fluid signal were regressed out as covariates. The ALFF was computed as the mean value of the fast Fourier transform of the voxel time series within a specific frequency domain (0.01–0.1 Hz). For T1‐weighted structural MRI data, a fully automatic analysis was performed using the SPM software. Firstly, the T1 images were segmented into gray matter and white matter images in their original space, then modulated and spatially normalized using the DARTEL toolbox (see Method S2 for details). Finally, the ALFF images and gray matter images were volumetrically smoothed using a Gaussian filter with a full width at half maximum (FWHM) size of 8 × 8 × 8 mm.

### 2.3 Extraction of Biomarker Information for SuStaIn Modeling

In this study, the ALFF was utilized as an fMRI biomarker to discern disease subtypes and elucidate their progression trajectories within the SuStaIn modeling framework. The ALFF metric encapsulates the synchrony and intensity of local rs‐fMRI activity within the brain, providing insights into the functional dynamics of the brain at rest. Initially, the AAL‐2 template served as a standardization platform to extract ALFF values from each of the 47 designated brain regions across participants. Notably, the ALFF value assigned to each brain region was derived by averaging the corresponding values from both the left and right hemispheres of that particular region, thereby ensuring consistency and comprehensiveness in our analysis.

To remove the influence of multisite variability, the Combat algorithm was implemented in our analysis (Fortin et al. 2018). To examine brain regions with ALFF abnormalities in MDD, we utilized a two‐tailed, two‐sample *t*‐test to contrast the mean ALFF values of the 47 brain regions between patients with MDD and HC, followed by sensitivity analyses with covariate adjustment (age, sex, education, and head motion). This comparison revealed 12 distinct brain regions exhibiting statistically significant differences (*p* < 0.05). Subsequently, a feature matrix was constructed, consisting of 833 patients with MDD across these 12 selected brain regions, to serve as input for the SuStaIn model. To address potential confounding variables, including age, gender, education level, and FD, a regression analysis was performed on the feature matrix. This step aimed to adjust for these factors, ensuring the robustness of our findings. Following adjustment, *z*‐scores of each variable were calculated for participants. This calculation involved subtracting the adjusted mean ALFF value of the HC group from the corresponding adjusted value observed in patients with MDD, and then dividing the resulting difference by the standard deviation of the HC group. Following prior research, we defined severity thresholds for the SuStaIn features based on *z*‐scores (Chen, Wang, et al. 2023). Specifically, *z*‐scores of 1 (indicating 1 standard deviation from the norm), 1.5, and 2 were adopted as severity thresholds, signifying increasing levels of disease progression for the considered brain regions. This approach enabled us to stratify patients based on their disease severity and explore disease trajectories within the SuStaIn modeling framework. Additionally, considering the potential association between functional and structural brain changes, during the feature selection stage, we regressed out the gray matter volume (GMV) from the ALFF values of the 47 brain regions and re‐ran the model for validation.

### 2.4 Data‐Driven Disease Progression Modeling Using SuStaIn

In this study, we adopt SuStaIn as a data‐driven disease progression model based on cross‐sectional MDD‐related ALFF changes. This framework aims to infer distinct disease subtypes and their stages along a constructed trajectory, thereby elucidating the intricate dynamics of disease evolution in a rigorous, data‐informed manner. SuStaIn's core comprises a linear *z*‐score model, which is fundamentally rooted in an event‐centric paradigm (Hong et al. 2023). This paradigm conceptualizes disease progression as a sequential unfolding of events, each marked by regional ALFF alterations transitioning from normative to pathological levels (Young et al. 2021). Through this approach, we endeavored to provide an insightful portrayal of the temporal and spatial evolution of the disease process.

**FIGURE 1:**
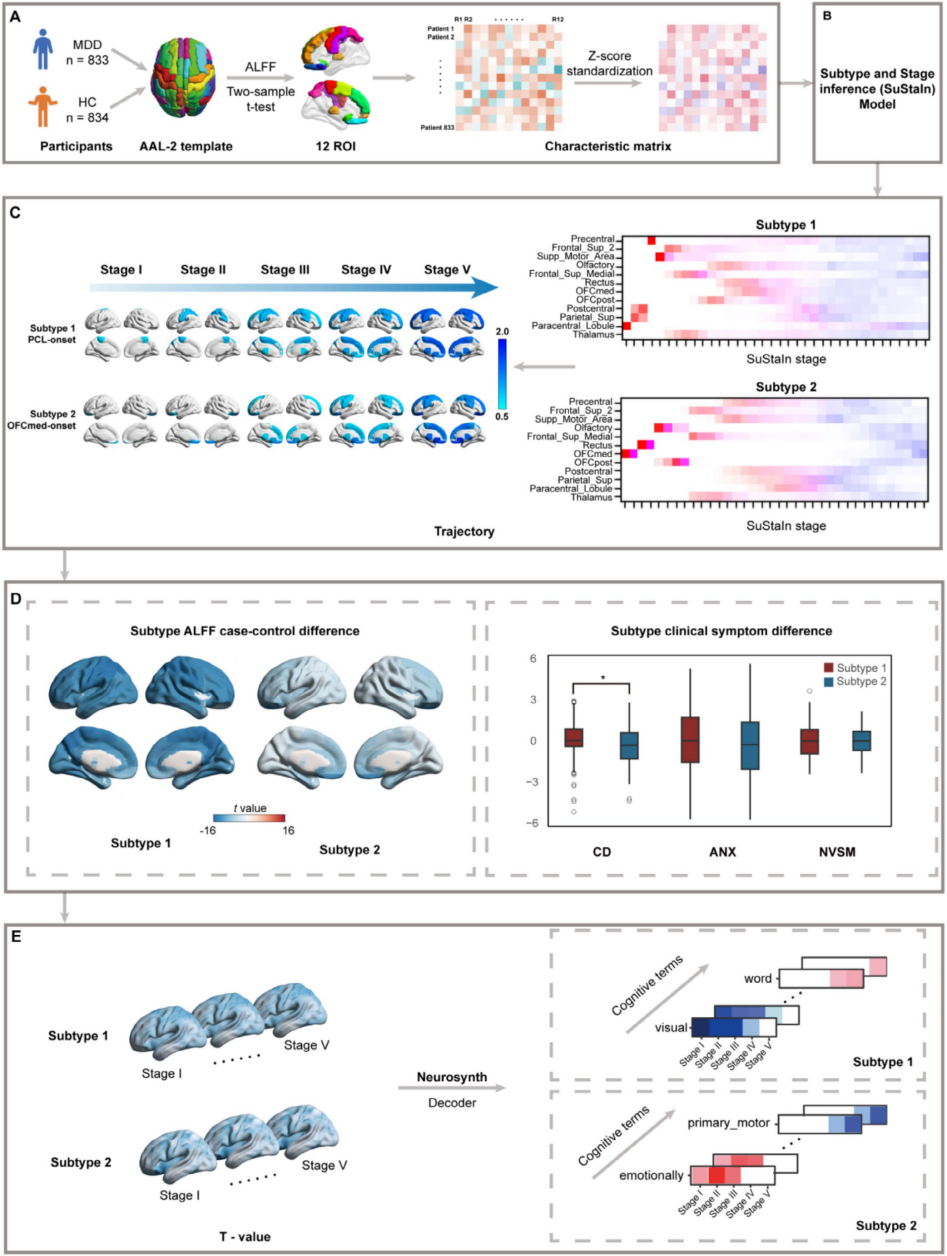
A conceptual overview of SuStaIn modeling. This schematic diagram provides an intuitive overview of the fundamental framework employed in this study. (A) Participant and feature matrix for SuStaIn; (B) data‐driven subtype and stage inference model (SuStaIn); (C) identification of MDD subtypes by SuStaIn model; (D) the comparison of clinical characteristics between the identified MDD subtypes, elucidating the distinctive features that differentiate them; (E) associations between *t*‐values at each stage of the two MDD subtypes and cognitive meta‐analytic activations. Abbreviations: ANX, anxiety; CD, core depression; NVSM, neurovegetative symptoms of melancholia.

A conceptual illustration of the SuStaIn modeling approach is presented in Figure 1. The analysis entailed 12 distinct variables, each representing changes in regional ALFF, and each of these variables was associated with three distinct *z*‐score severity levels (1, 1.5, and 2). Consequently, within each disease subtype, we identified a comprehensive set of 36 potential events (or stages) spanning from the earliest (Stage 1) to the latest (Stage 36). The vast array of potential progression patterns associated with these ALFF changes posed a significant challenge, rendering a comprehensive evaluation of every conceivable scenario impractical. To address this issue, we adopted the Markov Chain Monte Carlo (MCMC) sampling method, a computationally intensive yet powerful technique that approximates the posterior distribution through iterative simulations (Young et al. 2021). This approach allowed us to indirectly infer the underlying uncertainty in the progression patterns. The MCMC model was initialized using the expectation–maximization algorithm, a well‐established method for parameter estimation in statistical models. To ensure robustness, the process was repeated with 10 distinct random starting points, aiming to converge on the maximum likelihood solution. In determining the optimal number of disease subtypes, we implemented a five‐fold cross‐validation procedure on the SuStaIn data. Model performance was assessed using the cross‐validation information criterion (CVIC) and out‐of‐sample log‐likelihood, with lower CVIC scores and higher log‐likelihood values indicative of superior model fit and predictive accuracy (Chen, Wang, et al. 2023). This approach facilitated the identification of the most parsimonious and informative number of subtypes that best captured the underlying disease progression dynamics.

### 2.5 Associations Between Subtype‐Specific ALFF Alterations in MDD and Cognitive Meta‐Analytic Activations

We utilized Neurosynth (https://neurosynth.org/) to assess the relationship between meta‐analytically derived cognitive terms and alterations in whole‐brain ALFF across two distinct subtypes of MDD (Yarkoni et al. 2011). For patients with MDD stratified into different stage bins within these two subtypes, we initially performed between‐group comparisons of whole‐brain voxel‐wise ALFF, focusing on voxels that exhibited reduced ALFF compared to controls. These selected voxels were then used to generate *t*‐values for each stage. Subsequently, we employed the “decoder” function within Neurosynth to analyze the spatial correlations between these *t*‐values and the meta‐analytic maps corresponding to each term in the database. This approach allowed us to identify potential cognitive processes associated with the observed ALFF changes in patients with MDD across different stages and subtypes.

### 2.6 Statistical Analysis

A two‐tailed, two‐sample *t*‐test was implemented to examine the between‐group variations in demographic and clinical data. The chi‐squared test was applied to assess gender differences. Drawing upon previous research, we mapped the 17‐item Hamilton Depression Rating Scale (HAMD‐17) onto the National Institute of Mental Health's Research Domain Criteria (RDoC) framework, deriving three distinct clinical dimensions for MDD: core depression (CD), anxiety (ANX), and neurovegetative symptoms of melancholia (NVSM) (Ahmed et al. 2018). Each of these dimensions was subsequently compared between MDD subtypes using two‐tailed, two‐sample *t*‐tests, with age, gender, and education as covariates to mitigate potential confounding effects. A two‐tailed, two‐sample *t*‐test was performed to calculate the differences in GMV between MDD subtypes and HC across 47 brain regions (averaged across the left and right hemispheres), with gender, age, and education level controlled as covariates. Pearson correlation analysis was conducted to quantify the association between the age of onset and the total HAMD‐17 score for each MDD subtype. To ensure the robustness of our findings, the statistical significance threshold was set at *p* < 0.05, corrected for multiple comparisons employing the false discovery rate (FDR) method. This rigorous approach facilitates the interpretation of our results within the context of rigorous academic research standards.

## 3 Results

### 3.1 Subtype‐Specific Disease Progression Patterns

Compared to healthy controls, MDD exhibited a significant decrease (*p* < 0.05) in the ALFF within 12 distinct brain regions, including the precentral gyrus (PreCG); superior frontal gyrus, dorsolateral part (SFG); supplementary motor area (SMA); olfactory (OLF) cortex; superior frontal gyrus, medial part (SFGmedial); gyrus rectus (REC); medial orbitofrontal cortex (OFCmed); posterior orbitofrontal gyrus (OFCpost); postcentral gyrus (PoCG); superior parietal gyrus (SPG); paracentral lobule (PCL) and THA. These regions are mainly distributed in the somatomotor network (SMN), DMN, and limbic network (LIM). Results of the sensitivity analysis indicated that covariate‐adjusted regression identified 16 significant brain regions, showing substantial spatial concordance with the original 12 regions (Jaccard similarity = 0.75). Importantly, feature ranking stability remained high (Spearman's *ρ* = 0.86, *p* < 0.001), indicating that covariate effects on key features were well controlled.

**FIGURE 2:**
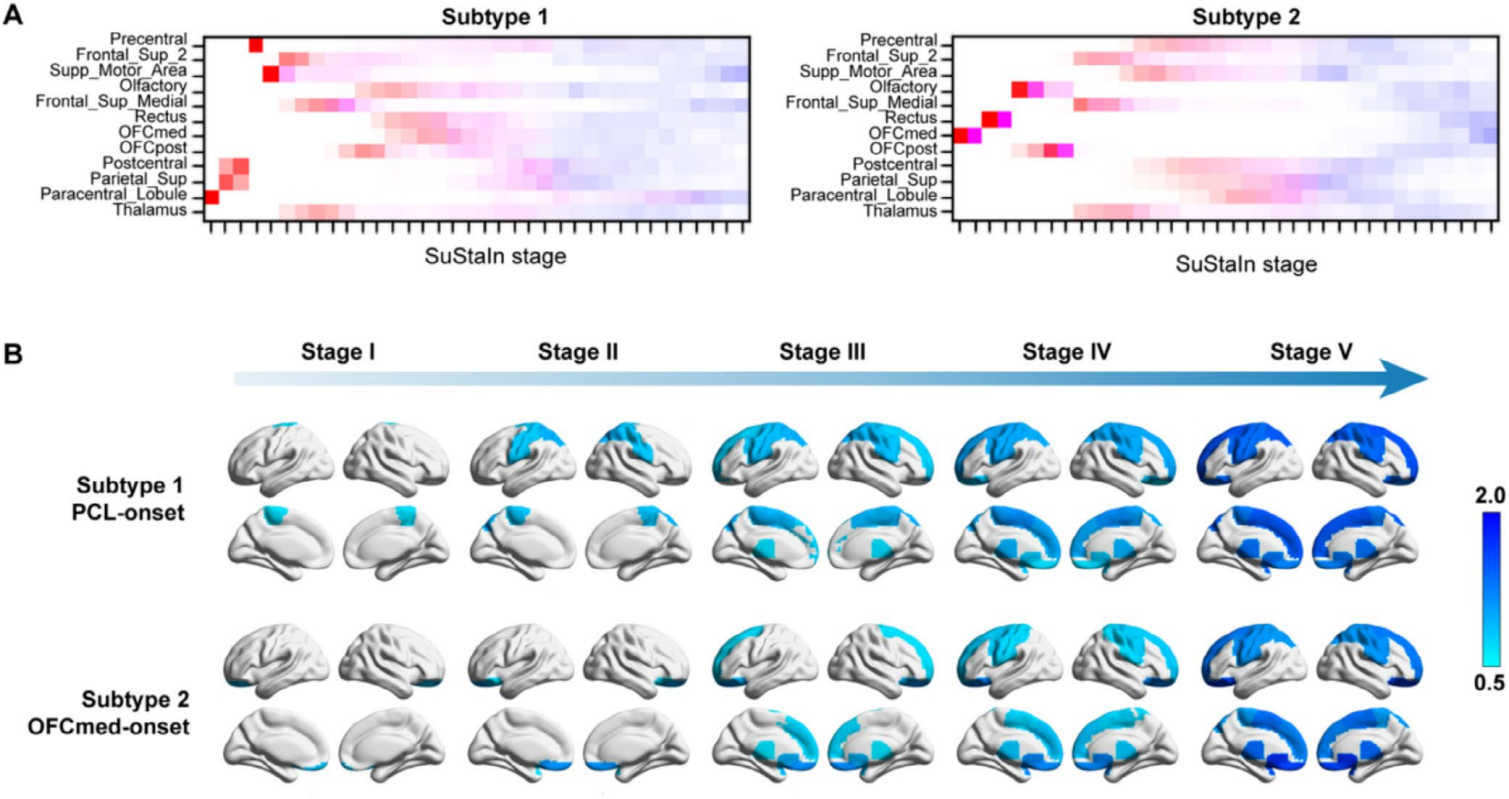
Developmental trajectories derived from SuStaIn analysis. (A) The position variance plot depicts the cumulative probability of individual brain regions attaining specific *z*‐score thresholds, color‐coded to signify the severity of ALFF reduction. Specifically, red indicates mild reduction (*z* = 1), magenta denotes moderate reduction (*z* = 1.5), and blue signifies severe reduction (*z* = 2). The color density reflects the proportion of posterior distribution events occurring at distinct positions within the sequence, as per the reference methodology (Chen, Wang, et al. 2023). (B) Visual depiction of ALFF abnormality progression across 36 stages in the SuStaIn model. This panel presents an intuitive illustration of the evolution pattern of ALFF abnormalities by calculating the average *z*‐score images for individuals grouped into distinct stage bins for the two subtypes: I (comprising Stage 1), II (Stages 2 and 3), III (Stages 4 and 5), IV (Stages 6 to 9), and V (Stages 10 and above). Each stage bin highlights brain regions with regional average *z*‐scores exceeding 0.65, providing a quantitative measure of ALFF abnormality progression.

To accurately assess the clustering tendency, we employed the CVIC and out‐of‐sample log‐likelihood analysis prior to conducting data‐driven subtyping. Both of these metrics are highly regarded for evaluating the quality of internal clustering, where higher values signify superior clustering performance. As depicted in Figure 2A, both indices attained their peak values at two clusters, conclusively indicating that the optimal number of clusters for k‐means clustering was *k* = 2. Based on this thorough analysis, we successfully identified two distinct trajectories of brain changes in MDD using the SuStaIn model, and subsequently classified patients with MDD into these distinct subtypes. As illustrated in Figure 2B, Subtype 1 exhibited a pattern of ALFF deviation that originated in the PCL, progressively spreading to the THA and ultimately affecting the OFCmed. Conversely, Subtype 2 displayed a completely opposing developmental trajectory, with ALFF initially decreasing in the OFCmed and gradually extending to the PCL. Notably, the characteristic progression patterns remained robust after statistically accounting for structural brain covariates (GMV regression), with detailed validation results presented in Supplemental Result S1.

**FIGURE 3:**
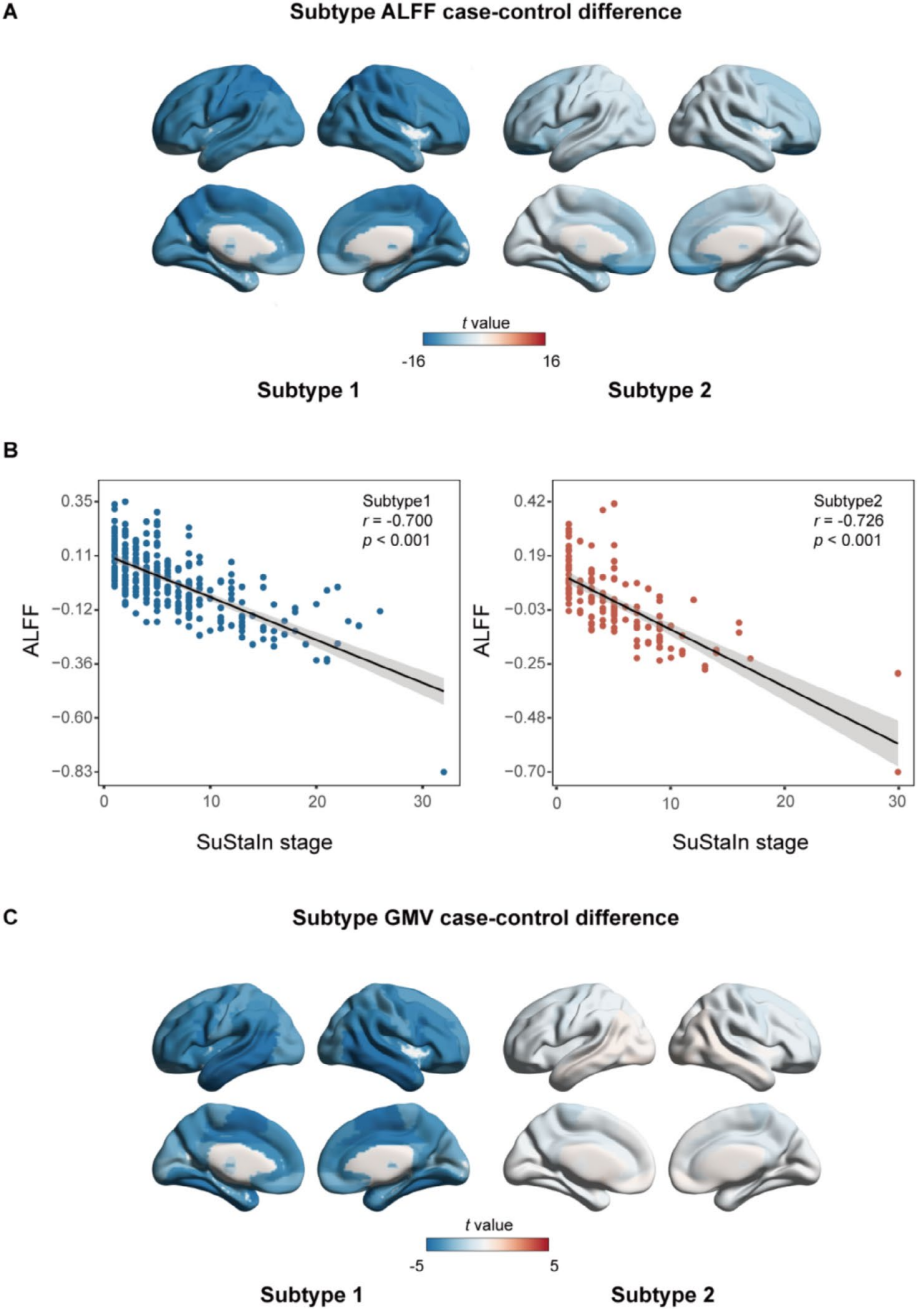
Subtype‐specific ALFF and GMV changes. (A) Regional ALFF differences among two subtypes and HC. Two‐sample *t*‐tests (two‐tailed, 47 times FDR‐corrected) were employed to assess differences in ALFF between the two subtypes and the HC group in each brain region. (B) Correlation between whole‐brain mean ALFF and SuStaIn stages across subtypes. The relationship between the mean ALFF of the entire brain and the SuStaIn‐derived disease stages was analyzed for both subtypes using Pearson correlation (two‐tailed *p* value reported). (C) Regional GMV differences among two subtypes and HC. Differences in GMV between the two subtypes and the HC group in each brain region were evaluated using two‐sample *t*‐tests (two‐tailed, 47 times FDR‐corrected).

As depicted in Figure 3A, both subtypes of MDD exhibited a significant reduction in ALFF across the entire brain when compared to healthy controls. A statistically significant negative correlation was observed between whole‐brain mean ALFF and developmental stage, as confirmed by Pearson correlation analysis. Specifically, for the subtype led by the PCL deviations, the correlation coefficient was *r*_subtype1_ = −0.700 with a *p* value of *p*_subtype1_ < 0.001. For the subtype led by the OFCmed deviations, the correlation coefficient was *r*_subtype2_ = −0.726 with a *p* value of *p*_subtype2_ < 0.001 (Figure 3B). Additionally, Subtype 1 displayed varying degrees of GMV atrophy in the brain, whereas Subtype 2 did not exhibit any statistically significant differences in GMV compared to the controls (Figure 3C).

### 3.2 Demographic and Clinical Characteristics of Two MDD Subtypes

The demographic and clinical profiles of these two subtypes are summarized in Table 1. No statistically significant differences were observed between the two MDD subtypes in age (*t* = −1.071, *p* = 0.285), gender distribution (*χ* ^2^ = 0.343, *p* = 0.558), total HAMD score (*t* = 0.979, *p* = 0.328), or duration of illness (*t* = −1.257, *p* = 0.210).

**TABLE 1:** Subtype‐specific demographic data.

	Subtype 1 (*n* = 294)	Subtype 2 (*n* = 138)	Group comparisons
	(Mean ± SD)	(Mean ± SD)	
Age (years)	36.065 ± 11.215	37.370 ± 12.982	*t* = −1.071, *p* = 0.285^a^
Sex (male/female)	96/198	49/89	*χ* ^2^ = 0.343, *p* = 0.558^b^
17‐item HAMD scores	22.429 ± 6.492	21.775 ± 5.297	*t* = 0.979, *p* = 0.328^a^
Duration of illness (months)	35.943 ± 57.291	43.880 ± 74.777	*t* = −1.257, *p* = 0.210^a^
Onset status (first‐episode/recurrent)	144/44	71/18	*χ* ^2^ = 0.351, *p* = 0.553^b^
Medication (used/not used)	95/93	46/43	*χ* ^2^ = 0.032, *p* = 0.858^b^

*Note:* Data on the duration of illness were available for 384 patients. Data on the onset status and medication status were available for 277 patients.

^a^ The *p* value was obtained by a two‐tailed two‐sample *t*‐test.

^b^ The *p* value was obtained by a chi‐squared test.

**FIGURE 4:**
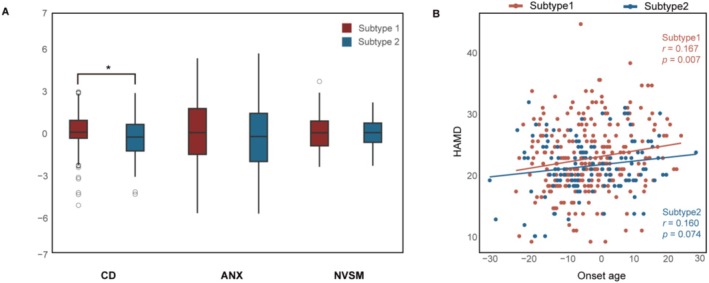
Distinct clinical characteristics across two MDD subtypes. (A) Comparison of clinical dimensions between the two MDD subtypes: Utilizing two‐sample *t*‐tests, we examined disparities in the domains of core depression (CD, comprising HAMD items 1 and 7), anxiety (ANX, encompassing HAMD items 9, 10, 11, and 15), and neurovegetative symptoms of melancholia (NVSM, including HAMD items 6 and 12). (B) Correlation between total HAMD score and age of onset.

Although no statistically significant difference was observed in the total HAMD score between the two subtypes, Subtype 1 demonstrated significantly higher scores on specific HAMD subscales (Figure 4A), notably the CD sub‐item compared to Subtype 2 (*t* = 2.470, *p*_FDR_ = 0.043, three times FDR‐corrected). No statistically significant differences were discerned in the remaining two HAMD subscales between the two subtypes (*p* > 0.05). Furthermore, across SuStaIn‐derived stages, both subtypes showed positive (though nonsignificant) correlations with CD scores (Subtype 1: *r* = 0.080, *p* > 0.05; Subtype 2: *r* = 0.070, *p* > 0.05). Subtype 1 exhibited a significant correlation between the age of onset and the total HAMD score (Figure 4B), as evidenced by a Pearson correlation coefficient of *r* = 0.167 (*p* = 0.007; Figure 4B). In contrast, no such significant correlation was detected in Subtype 2.

### 3.3 Meta‐Analytic Insights Into Brain Functions Associated With ALFF Alterations in MDD

**FIGURE 5:**
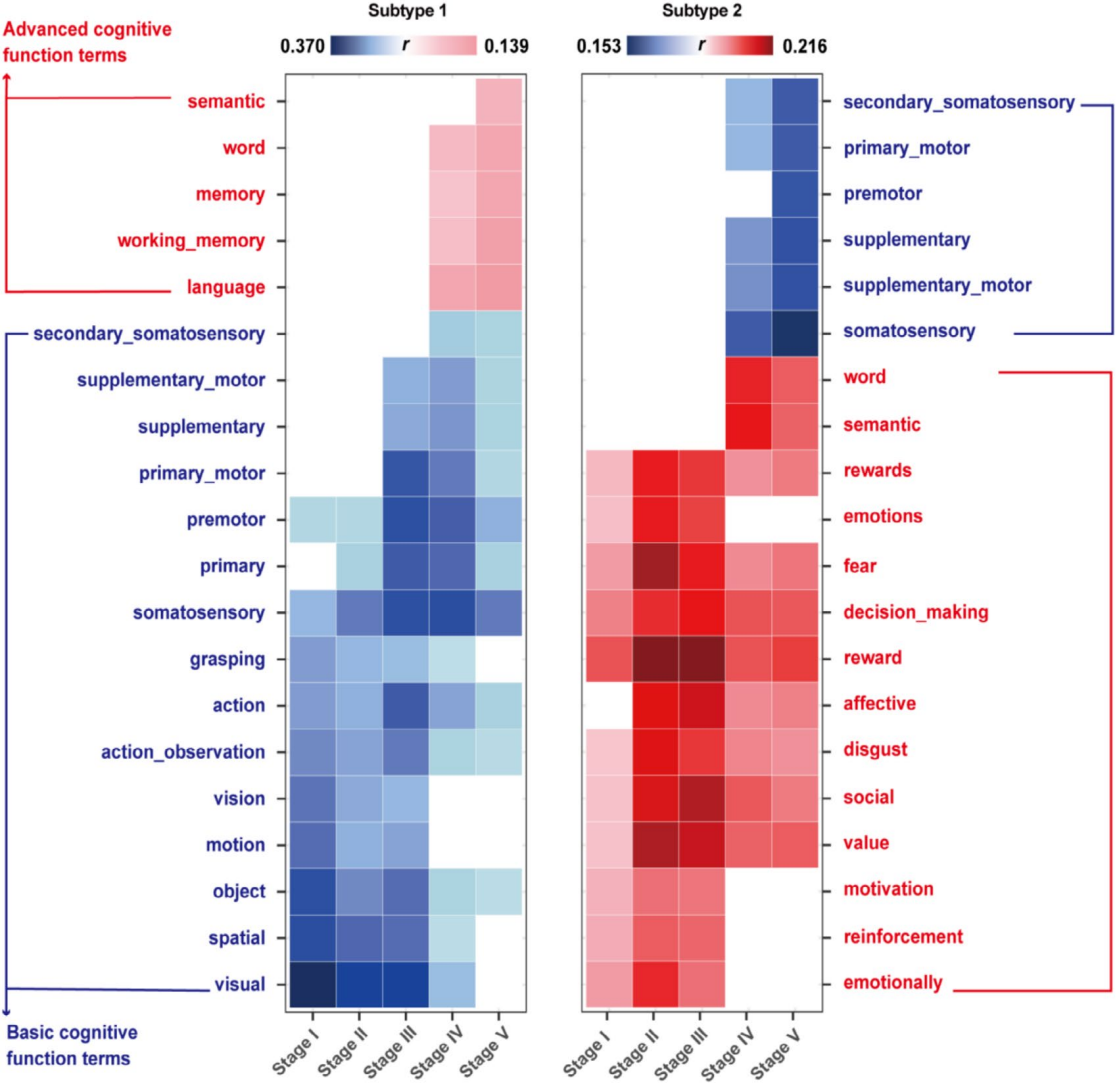
Association of two MDD subtypes with meta‐analytic cognitive terms. Spatial associations of whole‐brain ALFF reduction across stages in two subtypes decoded via Neurosynth. Blue represents cognitive terms related to simple cognitive functions, whereas red represents cognitive terms related to complex cognitive functions. The darker color intensity signifies a stronger correlation with the respective cognitive concept.

In Subtype 1 during the initial three stages, the brain regions exhibiting decreased ALFF were predominantly associated with meta‐analytic functional terms related to basic sensory and motor processes. These encompassed brain functions such as vision, spatial, and somatosensory. However, as we progressed to the subsequent two stages, a notable shift was observed. The cognitive terms associated with the affected regions shifted toward those related to higher‐order cognitive functions. Specifically, terms pertaining to memory and word gained prominence, suggesting a progression from basic to more complex cognitive impairments in Subtype 1 of MDD (Figure 5). This observed shift in Subtype 1 indicated a gradual attenuation in the correlation with terms indicative of fundamental brain functions, accompanied by a concurrent augmentation in the strength and prevalence of associations tied to higher‐order cognitive processes.

Conversely, Subtype 2 showed a distinct pattern, where the initial three stage bins exhibited robust correlations with meta‐analytic functional terms related to intricate emotional and value‐based processes. These correlations generally exhibited a declining trend over time. Remarkably, in the final two stages, meta‐analytic functional terms associated with somatosensory and primary motor functions re‐emerged, suggesting a potential reversal or modulation of the initially prominent brain functional profiles in Subtype 2. This finding underscores the heterogeneity and dynamic nature of brain alterations in different MDD subtypes.

## 4 Discussion

In this study, we employed a novel, data‐driven disease progression model known as SuStaIn to infer different and unique temporal progression patterns of altered ALFF in patients with MDD. Based on these trajectories, we distinguished the patients into two distinct subtypes, underscoring the inherent heterogeneity of the MDD population. Notably, one subtype was traced to originate from the PCL, whereas the other emerged from the OFCmed, emphasizing the distinct neurophysiological dynamics underlying these subtypes. The developmental trajectories of these two MDD subtypes were diametrically opposed, manifesting in clinically observable differences and correlating with varying degrees of abnormalities in brain functional activity. These findings underscore the imperative need to recognize the heterogeneity within the MDD population and suggest a potential requirement for the development of personalized therapeutic strategies tailored to the specific subtypes, ultimately enhancing the treatment efficacy for patients with MDD.

Subtype 1 of MDD originated in the PCL and exhibited a progressive trajectory, ultimately reaching the OFCmed. In contrast, Subtype 2 demonstrated an opposed trajectory, initiating in the OFCmed and subsequently extending toward the PCL. This divergence in alterations of ALFF underscored the distinct progression patterns of these two MDD subtypes. The PCL, situated proximal to the central sulcus, comprises two distinct regions: Its posterior segment, which constitutes the primary sensory cortex (S1) alongside the PoCG, and its anterior segment, which integrates with the PreCG to form the primary motor cortex (M1). S1 is intricately linked to somatosensory perception, empathy, and emotion regulation (Gao et al. 2023; Kropf et al. 2019), and prior studies have documented aberrant functional alterations in S1 among patients with MDD (Chen et al. 2021; Zhu et al. 2023), particularly those exhibiting somatic symptoms. Notably, patients with MDD with somatic symptoms exhibit significantly reduced ALFF in the PoCG and PreCG compared to those without somatic symptoms (Liu, Tu, et al. 2021). M1, which primarily receives somatosensory input from S1 (Gomez et al. 2021), is responsible for motor execution and learning, and impairments in motor cortex plasticity have been reported in MDD (Li et al. 2023). Stimulation of the motor cortex via repetitive transcranial magnetic stimulation (rTMS) has shown therapeutic potential in MDD (Hu et al. 2024). The OFCmed, in conjunction with the posterior orbitofrontal cortex (OFCpost) and OFL cortex, comprises the orbitofrontal cortex, a region vital for advanced cognitive functions encompassing learning, memory, emotion regulation, and social behavior (Rudebeck and Rich 2018). Existing research has revealed disrupted functional connectivity between the orbitofrontal cortex and various brain regions in MDD compared to healthy controls (Gong et al. 2020; Pizzagalli and Roberts 2022). Additionally, patients with MDD exhibited thinner orbitofrontal cortex and reduced surface area, underscoring structural abnormalities (Schmaal et al. 2017). Our findings identified two distinct MDD subtypes characterized by opposing ALFF reduction trajectories originating from PCL and OFCmed. These insights may inform the development of targeted interventions aimed at restoring brain region function and alleviating clinical symptoms in patients with MDD.

From a brain network perspective, our findings revealed two distinct MDD subtypes, each characterized by opposing developmental trajectories along a major functional gradient within the human brain. Subtype 1 originated from the SMN and progressed toward the DMN and LIM, whereas Subtype 2 initiated from LIM and DMN, ultimately evolving toward SMN. Previous studies have established that one extremity of the primary gradient in the human brain was anchored by the visual network (VIS) and SMN, with the opposite end anchored by DMN (Margulies et al. 2016). Similarly, MDD exhibited a gradient pattern transitioning from unimodal to transmodal networks (Xia et al. 2022). Our results demonstrated that both subtypes adhered to this major gradient but traversed it in opposing directions. The SMN, a crucial hub responsible for receiving and processing sensory inputs and coordinating motor functions (Sun et al. 2024), has been shown by previous research to be associated with abnormal functional connectivity changes in MDD (Grot et al. 2024; Liu, Fan, et al. 2021). These alterations may underlie the psychomotor retardation observed in patients with MDD (Buyukdura et al. 2011). In contrast, the LIM, identified through DCM, emerges as a common core abnormal network across psychiatric disorders (Ishida et al. 2023) and plays a pivotal role in the treatment response of patients with MDD (Li et al. 2022). The LIM, anchored in regions such as the amygdala, hippocampus, and anterior cingulate cortex, occupies a unique position in the functional hierarchy of the human brain. Although the primary functional gradient spans from unimodal (e.g., VIS, SMN) to transmodal (e.g., DMN) networks, LIM serves as a critical intermediary hub that bridges visceral‐autonomic processing (traditionally linked to unimodal functions) and higher‐order emotional–cognitive integration (transmodal functions) (Margulies et al. 2016). The DMN, intricately involved in introspection, self‐focus, emotional regulation, and motivation, has been extensively studied in the context of depression (An et al. 2024). Numerous investigations have suggested that DMN abnormalities contribute to the development of depression (Kaiser et al. 2015; Yan et al. 2019), with alterations in functional connectivity and topological properties of the DMN consistently observed (Scalabrini et al. 2020; Yang et al. 2021). Our findings, which highlighted the contrasting developmental trajectories of MDD subtypes along this critical functional gradient, offered novel insights into the neurobiological underpinnings of MDD and its heterogeneity.

In line with previous research (Han et al. 2023; Yuan et al. 2022), our study demonstrated that both MDD subtypes exhibited marked reductions in whole‐brain functional activity levels compared to healthy controls, with a discernible trend toward further decline as the disease progresses. Although numerous prior studies have documented abnormal changes in brain function in MDD that coincided with structural alterations (Myoraku et al. 2022; Zhang et al. 2018), our findings revealed a notable distinction: Only Subtype 1 exhibited significant GMV atrophy at the whole‐brain level. Furthermore, our analysis revealed a unique association in Subtype 1, where both the age of onset and HAMD total score are significantly correlated, suggesting a potential link between early onset and disease severity within this subtype. These findings underscored the heterogeneity of MDD and highlighted the need for tailored approaches to understanding and treating its diverse manifestations. Compared to Subtype 2, Subtype 1 manifested higher CD scores, specifically in Subscales 1 (*depressed mood*) and 7 (*work and interests*) of the HAMD, indicative of a more profound depressive mood and a more severe loss of interest clinically. Although the clinical scores‐stage correlations did not reach statistical significance, the positive trends between CD scores and neuroimaging stages suggest that ALFF abnormality progression may partially reflect worsening clinical symptoms. This preliminary finding justifies future studies with larger samples or multimodal approaches to verify imaging‐symptom relationships.

These results underscored the dynamic and heterogeneous nature of cognitive alterations in MDD, with distinct subtypes exhibiting varying trajectories of cognitive involvement as the disease progressed. These identifiable developmental patterns are related to changes in cognitive functions, as evidenced by Neurosynth‐decoded analyses. Specifically, Subtype 1 demonstrated a transition from simple to complex cognitive functions, indicating that as the disease advanced, reduced brain activity became increasingly correlated with more intricate cognitive processes. In contrast, Subtype 2 initially showed a correlation with higher‐order cognitive terms but gradually shifted towards impairments in basic functions. These contrasting patterns suggest that the onset and progression of cognitive and physical impairments in MDD may follow a temporal sequence closely linked to patterns of reduced brain activity. In Subtype 1 patients, the initial impact likely manifested in simple cognitive functions, subsequently progressing to impairments in more complex cognitive domains, reflecting a sequential degradation of specific neural networks. Conversely, Subtype 2 patients experienced early disruptions in higher‐order cognitive functions, which preceded the emergence of certain physical symptoms. These findings underscore the complexity and heterogeneity of MDD, necessitating tailored interventions sensitive to the unique developmental trajectories of each subtype. Such an approach could facilitate more effective and targeted treatments, addressing the specific cognitive and physical impairments experienced by individuals within different MDD subtypes.

Furthermore, our observations revealed a consistent intermediate role of the THA across the developmental trajectories of both subtypes. As a pivotal sensory conduction center, the THA is integral to diverse brain functions, encompassing sensory information processing, attention regulation, decision‐making, and memory consolidation (Roy et al. 2022). It serves as a hub, receiving direct sensory inputs and projecting to diverse functional cortices while also integrating inputs from the cortex (Sun, Liu, et al. 2023). Therefore, structural and functional abnormalities within the THA may contribute significantly to the somatic and psychological manifestations of MDD (Sun, Liu, et al. 2023; Bordes et al. 2020). Previous research underscored the THA's extensive connectivity with multiple brain networks, including the THA‐SMN, THA‐DMN, and THA‐LIM, with MDD exhibiting disruptions in the functional connectivity between the THA and these networks (Kang et al. 2018; Northoff et al. 2021; Yang et al. 2023). Additionally, alterations in the cortical–striatal–thalamic–cortical (CSTC) circuit have been reported across various psychiatric and neurological disorders, emphasizing the circuit's involvement in diverse pathological processes (Goodman et al. 2021; Ji et al. 2019; Liu, Chu, et al. 2023; Tsuchiyagaito et al. 2023). Given the aforementioned findings, we postulated that the THA assumed a pivotal role as a conduit in the reciprocal development of unimodal and transmodal regions within distinct subtypes of MDD. Specifically, it was hypothesized to bridge the two extremities of the major gradient, thereby facilitating communication and enhancing connectivity between these functionally distinct yet interconnected brain regions. Nevertheless, this hypothesis necessitates rigorous scrutiny and validation through comprehensive research endeavors, including the examination of its underlying neurobiological mechanisms and its implications for MDD pathogenesis and treatment.

## 5 Limitations of the Study

Several considerations are paramount in the interpretation of our findings. First, although the SuStaIn algorithm provides estimates of functional abnormality progression trajectories using cross‐sectional MRI data, further validation of these findings with longitudinal data are necessary. Additionally, due to the substantial computational demands of the SuStaIn method, a balance must be struck between computational cost and statistical robustness, requiring the number of input features to be kept within a certain limit. In this study, we were constrained to focusing on 12 brain regions that exhibited statistically significant differences between the patient and control groups to extract biomarker information. This limitation may restrict our ability to capture the intricate dynamic patterns underlying MDD progression, potentially overlooking a more comprehensive characterization of abnormal functional changes in the brain. At last, the compelling hypothesis advanced in this study, postulating the THA as a pivotal player in the developmental trajectory of MDD, necessitates robust empirical validation through extensive and exhaustive research endeavors. Such endeavors are crucial to firmly establish the THA's pivotal role in driving MDD progression and to elucidate its intricate functional interactions within the complex pathological landscape of this disorder.

## Author Contributions

Shao‐Wei Xue and Yuhong Zheng had full access to all of the data in the study and took responsibility. Concept and design: Shao‐Wei Xue, Yuhong Zheng, Peng Wang. Acquisition, analysis, or interpretation of data: All authors. Drafting of the manuscript: Yuhong Zheng, Shao‐Wei Xue. Critical revision of the manuscript for important intellectual content: Yuhong Zheng, Shao‐Wei Xue, Peng Wang, Jinghua Wang, Jinhui Wang. Statistical analysis: Yuhong Zheng, Peng Wang. Obtained funding: Jinghua Wang, Shao‐Wei Xue. Administrative, technical, or material support: Yuhong Zheng, Shao‐Wei Xue. Supervision: Shao‐Wei Xue, Jinghua Wang.

## Conflicts of Interest

The authors declare no conflicts of interest.

## Supporting information


**Data S1.** hbm70249‐sup‐0001‐Supinfo.

## Data Availability

The participant dataset is accessible upon reasonable request to the Rest‐meta‐MDD consortium (http://rfmri.org/REST‐meta‐MDD). The meta‐analysis maps of cognitive terms were obtained from Neurosynth (https://neurosynth.org/). Python (version 3.11.5) was employed to perform the SuStaIn algorithm (https://github.com/ucl‐pond). BrainNet Viewer (version 20191031) was used for ROI‐wise image visualization. All other analyses, including correlation analysis and *t*‐tests, were performed with MATLAB (version 2020a).
