# Supplementary material for: Elucidating Development Trajectories of Brain Functional Abnormalities in Major Depressive Disorder Utilizing a Data‐Driven Disease Progression Model
Source: Hum Brain Mapp. 2025 Jun 4;46(8):e70249. doi: 10.1002/hbm.70249 (PMC12136716; doi:10.1002/hbm.70249)
Supplement: Supplementary file 1 — Data S1. hbm70249‐sup‐0001‐Supinfo. [file HBM-46-e70249-s001.docx]

**Elucidating development trajectories of brain functional abnormalities in major depressive disorder utilizing a data-driven disease progression model**

*Supplemental Information*

Contents

[Supplemental Methods 2](#_Toc1096828675)

[Method S1. Introduction of REST-meta-MDD project 2](#_Toc399914877)

[Method S2. Structural MRI processing 2](#_Toc1893006276)

[Supplemental Results 3](#_Toc786250427)

[Result S1. GMV-corrected progression patterns of functional brain abnormalities 3](#_Toc1044046598)

[Supplemental Figures 5](#_Toc202292949)

[Figure S1. SuStaIn-modeled trajectories after GMV regression 5](#_Toc470980642)

[Figure S2. GMV differences between Subtype1 and HC arcoss Stages 6](#_Toc146927252)

# Supplemental Methods

## Method S1. Introduction of REST-meta-MDD project

This project aims to enhance the comparability and statistical power of multi-center data through standardized preprocessing protocols, addressing key challenges in MDD research, including limited sample sizes and heterogeneous analytical approaches. The initiative integrates resting-state functional MRI data (encompassing ALFF, ReHo, functional connectivity, voxel-mirrored homotopic connectivity [VMHC], and dynamic functional connectivity metrics) from 1,300 MDD patients and 1,128 healthy controls across 25 research cohorts from 17 Chinese hospitals. Comprehensive clinical and demographic information is provided to facilitate imaging-clinical correlation studies. Beyond functional imaging, the dataset includes T1-weighted structural images to enable gray matter volume and morphological analyses.

## Method S2. Structural MRI processing

First, the initial 10 volumes were discarded, and slice-timing correction was performed. Then, the time series of images for each subject were realigned using a six-parameter (rigid body) linear transformation. After realignment, individual T1-weighted images were co-registered to the mean functional image using a 6 degrees-of-freedom linear transformation without re-sampling and then segmented into gray matter (GM), white matter (WM) and cerebrospinal fluid (CSF). Finally, transformations from individual native space to MNI space were computed with the Diffeomorphic Anatomical Registration Through Exponentiated Lie algebra (DARTEL) tool.

To compare the differences in gray matter volume (GMV) between two subtypes and HC, we first resampled the gray matter images to the Automated Anatomical Labeling (AAL-2) template and applied volumetric smoothing using a Gaussian filter with a full width at half maximum (FWHM) of 8×8×8 mm. Next, we extracted the GMV from 47 brain regions (averaged across left and right hemispheres) for each participant. Age, gender, and education level were included as covariates and regressed out. Finally, we performed two-tailed, two-sample t-tests to compare the differences in brain volume between each subtype and the HC group.

# Supplemental Results

## Result S1. GMV-corrected progression patterns of functional brain abnormalities

During the feature selection stage, we regressed out the gray matter volume (GMV) from the ALFF values of 47 brain regions. The results showed that 11 brain regions still retained significant functional abnormalities (p < 0.05). Based on these 11 brain regions, we re-ran the SuStaIn model and still identified two subtypes (Figure S1), whose trajectory patterns were highly consistent with the original results: Subtype 1 extends from the paracentral lobule (PCL) to the orbitofrontal cortex, while Subtype 2 starts from the gyrus rectus (REC) and medial orbitofrontal gyrus (OFCmed) and eventually progresses to the PCL. This finding demonstrates that functional abnormalities are not merely driven by structural changes but have independent pathological significance.

To further elucidate the temporal relationship between functional and structural changes, we visualized the brain atrophy patterns across different stages of Subtype 1. As shown in Figure S2, gray matter atrophy primarily emerged in the middle to late stages (stages 4-5) of the trajectory progression, while functional abnormalities (reduced ALFF) were already significantly present in the early stages (stages 1-2). This temporal dissociation suggests that functional abnormalities may precede structural damage and could potentially drive gray matter atrophy through neuroplastic mechanisms.

# Supplemental Figures

## Figure S1. SuStaIn-modeled trajectories after GMV regression


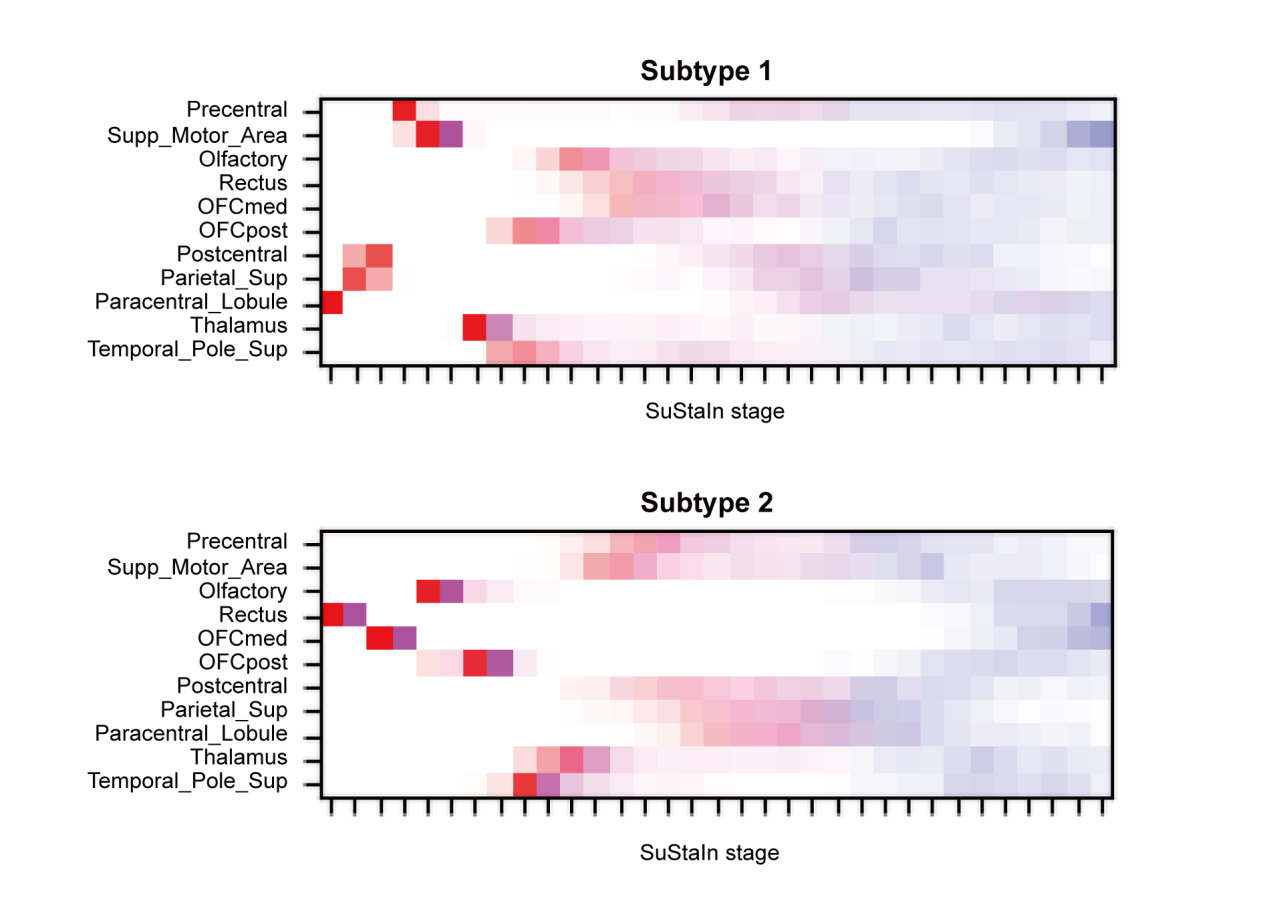


**Figure S1. SuStaIn-modeled trajectories after GMV regression.** The color density reflects the proportion of posterior distribution events occurring at distinct positions within the sequence.

## Figure S2. GMV differences between Subtype1 and HC arcoss Stages


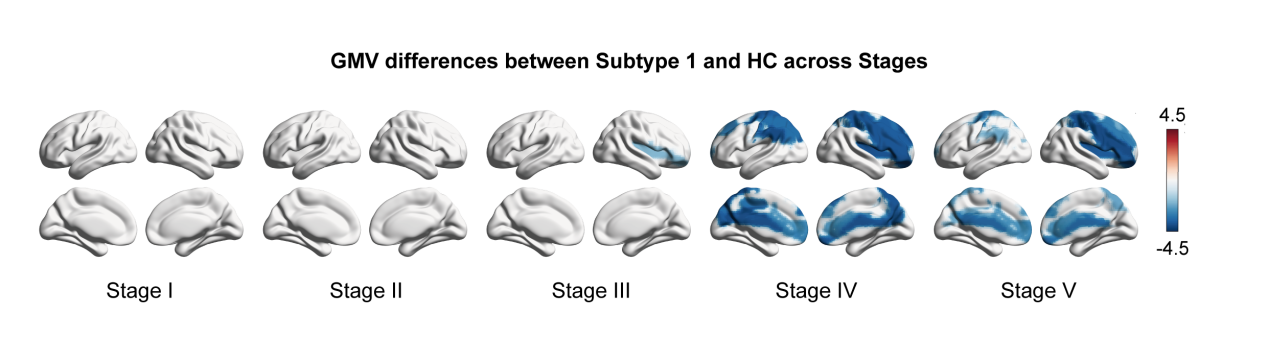


**Figure S2. GMV differences between Subtype1 and HC arcoss Stages.**
